# Estimating the Burden of Common Mental Disorders Attributable to Lifestyle Factors: Protocol for the Global Burden of Disease Lifestyle and Mental Disorder (GLAD) Project

**DOI:** 10.2196/65576

**Published:** 2025-03-14

**Authors:** Deborah N Ashtree, Rebecca Orr, Melissa M Lane, Tasnime N Akbaraly, Marialaura Bonaccio, Simona Costanzo, Alessandro Gialluisi, Giuseppe Grosso, Camille Lassale, Daniela Martini, Lorenzo Monasta, Damian Santomauro, Jeffrey Stanaway, Felice N Jacka, Adrienne O'Neil

**Affiliations:** 1 IMPACT (the Institute for Mental and Physical Health and Clinical Translation), Food & Mood Centre School of Medicine, Barwon Health Deakin University Geelong Australia; 2 Université Montpellier Institut National de Santé et de Recherche Médicale (INSERM) Desbrest Institute of Epidemiology and Public Health (IDESP) F-34090 Montpellier France; 3 IRCCS Neuromed Research Unit of Epidemiology and Prevention Pozzilli Italy; 4 Department of Medicine and Surgery Libera Università Mediterranea (LUM) University Casamassima (Bari) Italy; 5 Department of Biomedical and Biotechnological Sciences University of Catania Catania Italy; 6 ISGlobal Barcelona Spain; 7 Department of Medicine and Life Sciences Universitat Pompeu Fabra (UPF) Barcelona Spain; 8 CIBER Physiopathology of Obesity and Nutrition (CIBEROBN) Madrid Spain; 9 Division of Human Nutrition Environmental and Nutritional Sciences University of Milan, DeFENS-Department of Food Milan Italy; 10 Institute for Maternal and Child Health – IRCCS Burlo Garofolo Trieste Italy; 11 Queensland Centre for Mental Health Research Wacol Australia; 12 Faculty of Medicine School of Public Health University of Queensland Herston Australia; 13 Institute for Health Metrics and Evaluation University of Washington Seattle, WA United States; 14 Centre for Adolescent Health Murdoch Children's Research Institute Parkville Australia; 15 Department of Immunology, Therapeutics, and Vaccines James Cook University Queensland Australia

**Keywords:** mental health, depression, anxiety, diet, lifestyle, mental disorders, epidemiology, burden of disease

## Abstract

**Background:**

The Global Burden of Diseases, Injuries, and Risk Factors Study (GBD) collects and calculates risk-outcome data for modifiable lifestyle exposures (eg, dietary intake) and physical health outcomes (eg, cancers). These estimates form a critical digital resource tool, the GBD VizHub data visualization tool, for governments and policy makers to guide local, regional, and global health decisions. Despite evidence showing the contributions of lifestyle exposures to common mental disorders (CMDs), such as depression and anxiety, GBD does not currently generate these lifestyle exposure-mental disorder outcome pairings. This gap is due to a lack of uniformly collected and analyzed data about these exposures as they relate to CMDs. Such data are required to quantify whether, and to what degree, the global burden of CMDs could be reduced by targeting lifestyle factors at regional and global levels. We have established the Global burden of disease Lifestyle And mental Disorder (GLAD) Taskforce to address this gap.

**Objective:**

This study aims to generate the necessary estimates to afford the inclusion of lifestyle exposures as risk factors for CMDs in the GBD study and the GBD digital visualization tools, initially focusing on the relationship between dietary intake and CMDs.

**Methods:**

The GLAD project is a multicenter, collaborative effort to integrate lifestyle exposures as risk factors for CMDs in the GBD study. To achieve this aim, global epidemiological studies will be recruited to conduct harmonized data analyses estimating the risk, odds, or hazards of lifestyle exposures with CMD outcomes. Initially, these models will focus on the relationship between dietary intake, as defined by the GBD, and anxiety and depression.

**Results:**

As of August 2024, 18 longitudinal cohort studies from 9 countries (Australia: n=4; Brazil: n=1; France: n=1; Italy: n=3; The Netherlands: n=3; New Zealand: n=1; South Africa: n=1; Spain: n=1; and United Kingdom: n=3) have agreed to participate in the GLAD project.

**Conclusions:**

Our comprehensive, collaborative approach allows for the concurrent execution of a harmonized statistical analysis protocol across multiple, internationally renowned epidemiological cohorts. These results will be used to inform the GBD study and incorporate lifestyle risk factors for CMD in the GBD digital platform. Consequently, given the worldwide influence of the GBD study, findings from the GLAD project can offer valuable insights to policy makers worldwide around lifestyle-based mental health care.

**International Registered Report Identifier (IRRID):**

DERR1-10.2196/65576

## Introduction

Mental disorders rank among the top 10 leading causes of disease and economic burden worldwide. They account for 4.9% of global disability-adjusted life years [1] and it has been projected that by 2030 mental disorders will account for more than half of the economic burden attributable to noncommunicable diseases [2]. Given the recognized burden on individuals, communities, and economies [1,3], identifying risk factors for common mental disorders (CMDs), namely depression and anxiety disorders, is critical for early prediction, prevention, and treatment. While known risk factors such as genetics, childhood adversity, and family conflict have a significant impact on CMD risk [4], preventative efforts should also address potentially more modifiable factors that may have broader reach.

Over the past decade, evidence has emerged highlighting lifestyle behaviors as modifiable risk factors for CMDs. A recent meta-review, drawing from top-tier data such as meta-analyses of prospective cohort studies, Mendelian randomization studies, and randomized controlled trials, emphasized the significant role of lifestyle behaviors pertaining to diet quality, physical activity, smoking, and sleep in CMD risk [5]. Furthermore, current evidence indicates that targeting these lifestyle behaviors can ameliorate the risk of CMDs. For example, data involving adult participants from France estimate that 14% of incident cases of depression are attributable to a combination of unhealthful diet, weight, and smoking [6]. Promisingly, data from Norwegian adults show that 12% of de novo depression could be averted if an individual engages in at least 1 hour of physical activity per week [7]. Collectively, this evidence highlights the importance of lifestyle factors in mental health outcomes, which has implications regarding prevention and treatment.

Despite ample evidence supporting the inclusion of lifestyle-based mental health care in clinical recommendations and population-level strategies [8], exemplified by clinical practice guidelines from organizations such as the Royal Australian and New Zealand College of Psychiatrists [9], the global implementation of these strategies for addressing CMDs remains limited. Global nutritional recommendations focus largely on individual food groups or constituents, and not on the dietary patterns, such as the Mediterranean diet, that are commonly used in research. As such, the evidence regarding whether dietary components would be useful to the target population's mental health is unclear. Furthermore, it is widely acknowledged that numerous structural barriers hinder the improvement of lifestyle behaviors, including social, financial, and environmental determinants [10,11]. These barriers also exist in mental health treatment and care [12]. Therefore, there is merit in attempting to reduce the incidence of CMDs by improving lifestyle behaviors, not just at the individual level, but also through population and policy-level strategies. To inform such strategies worldwide, rigorous global data are required to quantify the contribution of lifestyle behaviors to the risk of population CMDs.

The largest epidemiological study worldwide, the Global Burden of Diseases, Injuries, and Risk Factors Study (GBD), provides important global health estimates that inform public health priority setting and policies. A critical tool used by the GBD to communicate the current state of health and disease for different countries is the digital platform, the GBD VizHub Data visualization tool [13]. This digital platform allows policy makers to quickly ascertain where the greatest improvements in public health could be achieved by targeting specific risk factors. The GBD currently provides comprehensive estimates of lifestyle risks, such as diet, for a range of physical conditions, but has yet to incorporate estimates of these lifestyle risks for CMDs. This may be due to challenges in operationalizing and standardizing the measurement of lifestyle factors and CMD outcomes across studies, aligning them with GBD classifications and definitions, and establishing clear cause-and-effect relationships, particularly given the typical clustering of lifestyle risk factors [14-16]. In addition, studies investigating the associations between lifestyle risk factors and CMDs often face issues such as a lack of global representation, small sample sizes, inconsistent methodologies, residual confounding, and measurement error, particularly in regard to measuring dietary intake or ensuring adherence in intervention studies [17]. Consequently, while these factors are well-recognized risk factors for physical illnesses like heart disease and diabetes, the potential for lifestyle targets to be considered relevant to the population’s mental health has historically been neglected [18].

To include lifestyle exposures as risk factors for CMDs on the GBD digital platform, the Global burden of disease Lifestyle And mental Disorder (GLAD) Taskforce (a large-scale, coordinated, international collaborative effort) has been formed. The Taskforce comprises global epidemiological experts who will work with GBD representatives to oversee the comprehensive evaluation of the association of lifestyle risk factors with CMDs, starting with dietary intake as the exposure of interest (the GLAD project). These experts have developed a harmonized approach for individual studies to conduct analyses (the GLAD project) to enable the integration of lifestyle factors as a risk for CMDs in the GBD. This paper outlines the processes by which the GLAD project and participant member studies will execute this harmonized data analysis protocol on their own datasets and disseminate their results through peer-reviewed publications. The overarching aim of the GLAD project is to generate robust, comparable evidence on the global risk of CMDs attributable to lifestyle factors, ultimately enabling their inclusion as risk factors for CMDs within the GBD framework. We hypothesize that more healthful lifestyle factors, such as higher intakes of healthful dietary components (eg, fruits, vegetables, whole grains, and fiber) will be associated with a lower risk of CMDs. Conversely, we hypothesize that less healthful lifestyle factors, such as higher intake of less healthful dietary components (eg, processed meat, sugar-sweetened beverages, and sodium) will be associated with a higher risk of CMDs.

## Methods

### Recruitment

**Figure 1:**
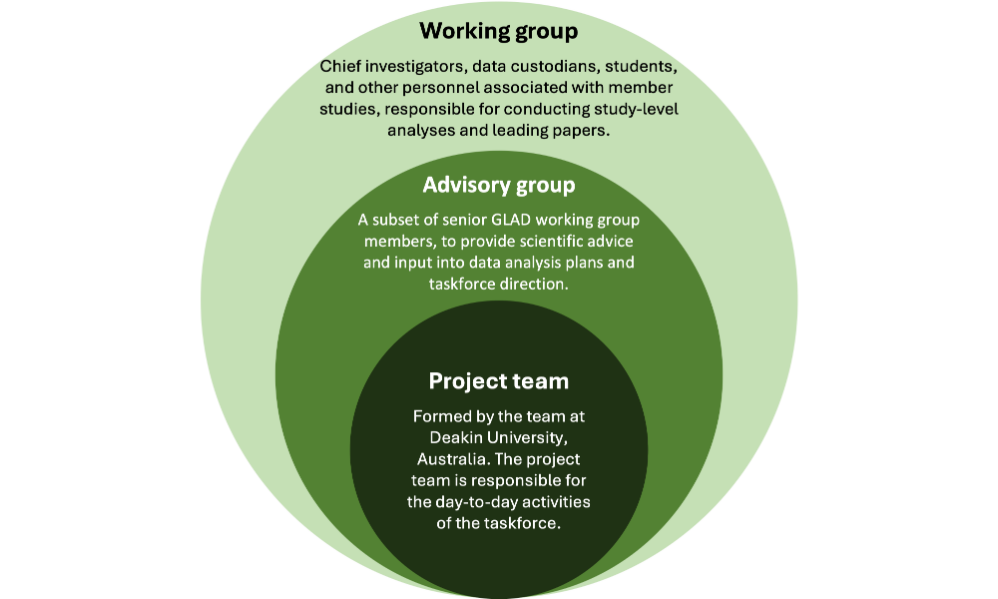
The GLAD taskforce composition and responsibilities within the GLAD project. GLAD: Global burden of disease Lifestyle And mental Disorders.

The GLAD project is a collective, multistudy initiative led by Deakin University’s, Food and Mood Center, which is situated within the Institute for Mental and Physical Health and Clinical Translation, School of Medicine, Barwon Health, Geelong, Australia. The GLAD project will be completed by the GLAD task force, comprising the project team, the advisory group, and the working group containing all member studies (Figure 1).

To identify potential member studies for participation in the GLAD project, which would apply the harmonized data analysis protocol to their own datasets and contribute their results to the broader initiative, we used a multistep process. The first step was to identify epidemiological studies that had the necessary variables by which the study lead or representative could execute our analysis plan (eg, food frequency questionnaires from which to derive GBD-defined dietary risks combined with mental health questionnaires to identify cases of depression or anxiety). This process involved a literature search to identify potentially eligible studies and key contacts to approach for invitation to participate in the GLAD project. To operationalize the dietary risk variables, we used previous GBD papers, which contained the definitions of each dietary exposure (Table 1) and depression and anxiety (Table 2) [1,19]. In addition to using the literature to identify potentially eligible datasets, we also drew on previous collaborations of the GLAD project team and researchers in the field of nutritional psychiatry (eg, via the International Society of Nutritional Psychiatric Research), as well as promoting the project on Food and Mood Center social media sites. Data custodians of identified studies were contacted, and those with relevant data and who had the capacity to participate in the GLAD project completed an expression of interest via the Food and Mood Center website.

To be eligible for inclusion, member studies must have dietary intake (Table 1) and depression and anxiety outcomes (Table 2) consistent with GBD definitions, or the ability to recode existing variables accordingly. Any deviations to these definitions must be reported and will be accounted for accordingly as reported below in “Future Directions: Meta-analyses.” Any study design, in any location, with any sample size, is eligible to be a member study and can complete the required data analysis.

**Table 1:** Dietary exposure definitions and required coding for the GLAD project^a^.

Dietary risk factor	GBD^b^ exposure definition	Required coding^c^
Fruits	Average daily consumption of less than 310-340 g of fruit including fresh, frozen, cooked, canned, or dried fruit, excluding fruit juices and salted or pickled fruits.	grams/day
Vegetables	Average daily consumption of less than 280-320 g of vegetables, including fresh, frozen, cooked, canned, or dried vegetables and excluding legumes and salted or pickled vegetables, juices, nuts and seeds, and starchy vegetables such as potatoes or corn.	grams/day
Legumes	Average daily consumption of less than 90-100 g of legumes and pulses, including fresh, frozen, cooked, canned, or dried legumes.	grams/day
Whole grains	Average daily consumption of less than 140-160 g of whole grains from breakfast cereals, bread, rice, pasta, biscuits, muffins, tortillas, pancakes, and other sources.	grams/day
Nuts and seeds	Average daily consumption of less than 10-19 g of nuts and seeds.	grams/day
Milk	Average daily consumption of less than 360-500 g of milk including non-fat, low-fat, and full-fat milk, excluding plant derivatives.	grams/day
Red meat	Any intake of red meat including beef, pork, lamb, and goat but excluding poultry, fish, eggs, and all processed meats.	grams/day
Processed meat	Any intake of meat preserved by smoking, curing, salting, or addition of chemical preservatives.	grams/day
Sugar-sweetened beverages	Any intake of beverages with ≥50 kcal per 226.8-g serving, including carbonated beverages, sodas, energy drinks, and fruit drinks, but excluding 100% fruit and vegetable juices.	grams/day
Fiber	Average daily consumption of less than 21-22 g of fiber from all sources including fruits, vegetables, grains, legumes, and pulses.	grams/day
Calcium	Average daily consumption of less than 1.06-1.1 g of calcium from all sources, including milk, yogurt, and cheese.	grams/day
Seafood omega-3 fatty acids	Average daily consumption of less than 430-470 mg of eicosapentaenoic acid and docosahexaenoic acid.	milligrams/day
Polyunsaturated fatty acids	Average daily consumption of less than 7%-9% of total energy intake from polyunsaturated fatty acids.	total percentage of daily energy intake
Trans fatty acids	Any intake of trans fat from all sources, mainly partially hydrogenated vegetable oils and ruminant products.	total percentage of daily energy intake
Sodium	Average 24-hour urinary sodium excretion greater than 1-5 g.	grams/day
Ultra-processed foods (optional)	Any intake of ultraprocessed foods, as defined by the Nova system as any food item in Nova category 4 (“ultraprocessed food”), in grams [20].	grams/day

^a^Based on the GBD (Global Burden of Diseases, Injuries, and Risk Factors Study) risk exposure definitions (Pages 217-218 in Supplementary Appendix 1 in the study by the the GBD 2019 Risk Factors Collaborators [19]).

^b^GBD: Global Burden of Diseases, Injuries, and Risk Factors Study.

^c^Analyses using continuous exposures are required by the GLAD Taskforce. The units listed here are recommended, however, these may be rescaled to more meaningful units in papers (eg, per 10 g) provided the original units are available to the project team.

**Table 2:** Mental health definitions and recommended coding for the GLAD^a^ project^b^.

Mental disorder	Outcome definition	Diagnostic reference	Recommended coding^c^
Major depressive disorder	Involves the presence of at least one major depressive episode, which is the experience of either depressed mood or loss of interest/pleasure, for most of every day, for at least two weeks.	DSM-IV-TR^d^: 296.21–24; 296.31–34. ICD-10^e^: F32.0-9; F33.0-9.	0=no MDD^f^; 1= MDD
Anxiety disorders (any subtype)	Involves experiences of intense fear and distress, typically in combination with other physiological symptoms. Anxiety disorders will be modeled as a single cause for “any” anxiety disorder to avoid the double-counting of individuals meeting criteria for more than one anxiety disorder. Epidemiological estimates reporting an outcome for “any” or “total” anxiety disorders will be included if they reported on at least three anxiety disorders.	DSM-IV-TR: 300.0-300.3; 208.3; 309.21; 309.81. ICD-10: F40-42; F43.0; F43.1; F93.0-93.2; F93.8.	0=no anxiety disorder; 1=anxiety disorder

^a^GLAD: Global burden of disease Lifestyle And mental Disorders.

^b^The definitions and diagnostic references presented in this table are the current definitions used by the GBD (Page 4 in the Supplementary Appendix in the study by the GBD 2019 Mental Disorders Collaborators [1]). Since GLAD members may use alternative methods to ascertain disorder status, definitions or diagnostic references may vary slightly. For example, somatic forms of depression may be captured more by symptom scales, or studies using prescriptions may use Anatomical Therapeutic Chemical codes to determine incident depression and anxiety.

^c^Outcomes must be binary (condition present vs condition absent). For studies that only use symptom scales, validated cutoffs can be used to determine condition present versus condition absent (refer to Multimedia Appendix 1).

^d^*DSM-IV-TR*: *Diagnostic and Statistical Manual of Mental Disorders (Fourth Edition, Text Revision)*.

^e^*ICD-10*: *International Statistical Classification of Diseases, Tenth Revision*.

^f^MDD: major depressive disorder.

### Statistical Analysis

#### Overview

The GLAD project and the following methods have been prospectively registered on the Open Science Framework [21]. The methods outlined herewith are based on the most up-to-date definitions used by the GBD, at the time of writing this protocol. Given that the GBD is subject to change, future iterations may vary in accordance with such amendments. The initial iteration of the GLAD project will focus on dietary exposures, and the methods described within this protocol refer specifically to this first iteration. Subsequent updates to the protocol may be made when additional lifestyle risk factors are considered in future iterations of the GLAD project.

#### Exposure and Outcome Definitions

Definitions of dietary intakes and CMDs are based on definitions used by the GBD [1,18]. In addition to the 15 dietary intakes used by the GBD, ultraprocessed food intake will be included where practicable given the increasing literature in the field linking this dietary exposure to a wide range of health outcomes [22-25]. In doing so, we will use the most widely adopted food classification system based on the purpose and extent of industrial food processing, Nova, to define ultraprocessed foods [20]. Furthermore, we will follow best practice guidelines when categorizing foods according to the Nova food classification system [26].

CMDs can be measured differently across epidemiological studies. They are most commonly assessed using self-reported symptom scales with cutoffs for diagnosis, from medical records, or using diagnostic interviews. Multimedia Appendix 1 outlines some commonly used tools and their recommended cutoffs for identifying symptoms of depression and anxiety. The sensitivity, specificity, and diagnostic accuracy of these tools largely depend on the population. When undertaking meta-analyses, we may conduct a sensitivity analysis to determine if the magnitudes of associations change based on exposure and outcome measurement or ascertainment type.

#### Covariates and Confounders

To address the anticipated heterogeneity of member studies’ available data, a set of core confounders are provided for the purpose of generating a consistent minimally adjusted analysis. The conduct of additional analyses (eg, using additional variables, fitting sensitivity models, or exploratory analyses) is at the discretion of the study author or authors based on available data and reviewer requests when studies are submitted for publication.

To be included in the GLAD project, member studies must adhere to the iteratively adjusted models (Textbox 1).

**The iteratively adjusted statistical models required for the Global burden of disease Lifestyle And mental Disorders (GLAD) task force.**

- Model 1: Unadjusted model, with the dietary variable (as listed in Table 2) as the exposure and depression or anxiety as the outcome.
- Model 2: Minimally adjusted model, with the dietary variable of interest, depression or anxiety, and with baseline mental disorder status (or lifetime history of mental disorders for cross-sectional studies), age, sex, and a measure of socioeconomic status, such as household income, employment status, or education.
- Sensitivity model: Same as model 2, but with adjustment for total energy intake.

Of particular relevance in nutritional epidemiology are methods for adjusting for energy intake. Intake of specific nutrients are correlated with total energy intake, and so appropriate adjustment is required to disentangle the effect of the nutrient from the effect of energy. For example, an individual may alter intake of a specific nutrient by changing dietary composition, and not by changing total energy intake. As such, controlling energy intake can reduce confounding, and failure to appropriately control for total energy intake may nullify associations between nutrient intake and disease outcomes [27]. Given the correlation between nutrient intake and energy, the application of Willett’s residual method is recommended to account for total energy intake [27]. This involves fitting a regression model with the dietary variable as the outcome and total energy intake as the exposure, predicting residuals from this model, and then using these predicted residuals in the analysis models [27].

#### Reporting Baseline and Demographic Characteristics

To describe participant characteristics, authors of member studies include a table containing descriptive statistics, given as n (%) for categorical variables, and mean (SD) or median (25th-75th percentile) for continuous variables. A column containing *P* values will not be included, as statistical tests do not meaningfully assess differences between samples or populations, nor should they be used as the basis for assessing confounders [28].

#### Estimating Risk Ratios, Odds Ratios, and Hazards Ratios

**Figure 2:**
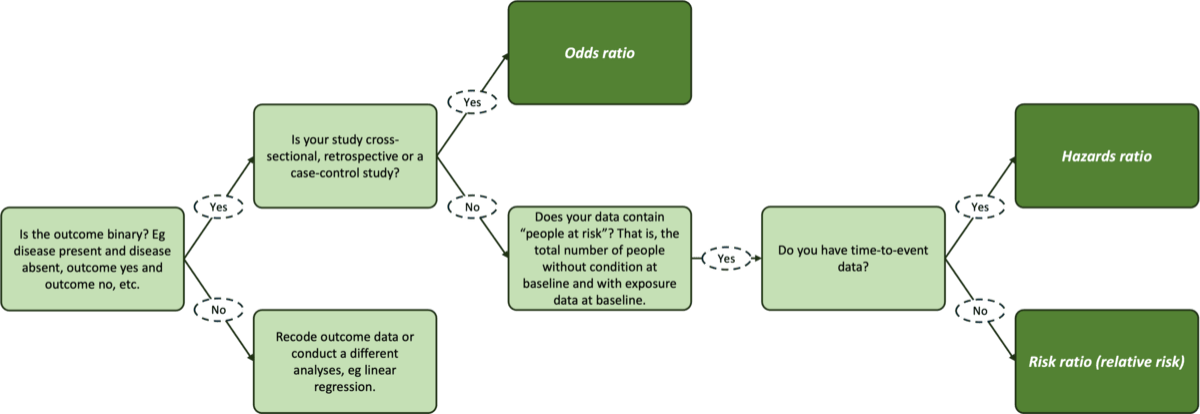
The types of ratio measures that can be obtained from binary outcome data. Odds ratios will be estimated using logistic regression, risk ratios will be estimated using Poisson regression with robust SE, and hazard ratios will be estimated using Cox regression.

Each study is required to use risk ratios (estimated by Poisson regression with robust SE) as the primary means of assessing the association of dietary intake with CMDs. Risk ratios are based on incident cases, and so models will need to exclude participants with depression or anxiety at baseline. Furthermore, to determine incident risk, studies must have dietary exposures at baseline and depression, or anxiety measured at a subsequent time point. Where the estimation of a risk ratio is not possible (refer to Figure 2), for example, where the total number of people exposed at baseline is not available (such as for retrospective or case-control studies) [29], odds ratios are estimated using standard logistic regression techniques. Where only time-to-event data are available, hazard ratios are used, estimated by a Cox proportional hazards model. For rigor, all effect estimates will be accompanied by a 95% CI and exact *P* value [30].

Models can be fitted using any statistical analysis software, including R (R Core Team), SAS/STAT (SAS Institute Inc), Stata (StataCorp LLC), and SPSS (IBM Corp). Before fitting the relevant models, Member studies must assess model assumptions. Should any model assumptions be violated, an alternate approach to analysis may be selected. For example, the nonlinearity of continuous variables should be investigated, and transformations or nonlinear models fitted where necessary. Similarly, should any of the assumptions of Cox proportional hazards models be violated, alternative strategies will be deployed to account for these violations.

In addition to the models listed above, the following subgroups shall be fitted separately for each exposure-outcome pairing, where data allows: (1) sex, (2) age, (3) year (for longitudinal studies with multiple waves of follow-up), (4) country (for multicenter studies), and (5) all symptom scales and diagnostic measures (for studies with depression and anxiety measured via multiple measures. For age, studies should explore the relationship between the risk factors and mental disorders with age to see if a continuous measure is appropriate. Where a continuous measure is not appropriate, studies should generate appropriate age categories for their dataset by inspecting the frequency of outcomes by age groups. As a guide, the GBD currently uses 0-6 days (early neonatal), 7-27 days (late neonatal), 28-365 days, 1-4 years, 5-9 years, then 5-year intervals from 10-95 [31].

### Sensitivity Models

#### Multiple Testing

Given the need for multiple models to be fitted within each study, the GLAD Taskforce recommends a *P* value adjustment procedure, such as the Simes method, to minimize the chance of a type 1 error and determine whether multiple testing may be influencing results [32]. This should be applied to all models in a study where multiple models are required.

#### Outliers and Influential Data

Initial models include all available data points, with sensitivity analyses included for any influential or outlying observations. Influential observations are determined by predicting residuals, Cook’s distance, or DFBETA after fitting the relevant model. Although several cutoff points have been proposed to identify influential observations, conventional cutoffs are applied: 3 for standardized residuals [33]; 4/(n-p) for Cook’s Distance [34], where n=sample size and p=number of parameters in the model; and 2/√n for DFBETA [35].

#### Missing Data

Each study initially uses a complete-case model, whereby individuals with missing observations are excluded from the model. Member Studies with missing exposure, outcome, and covariate observations conduct a missing data analysis to determine the influence of missing data. While a multiple imputation approach is preferred, other methods such as k-nearest neighbor imputation or inverse probability weighting may be used where an imputation approach is not possible or appropriate [36-41].

### Future Directions: Meta-Analyses

Upon completion of the analysis phase of the GLAD project (phase 2), the GLAD project team and advisory group will lead a meta-analysis (phase 3). A separate protocol will be created for this phase of the GLAD project. Briefly, the GLAD project team will conduct a systematic literature review to identify any relevant studies not already participating in GLAD. After screening abstracts and full text, data will be extracted from all identified studies and studies participating in GLAD. A random effects meta-analysis will be performed to pool results from all eligible studies. We will perform a meta-regression to quantify how the risk can vary by demographic factors, and methodological biases [42]. Where studies have used different assessment methods for mental health outcomes, different tools to ascertain dietary intake, any slight deviations to definitions, or where studies use different analysis strategies, we will perform cross-walking or sensitivity analyses to ensure comparability [43]. Papers using methods that deviate substantially from the methods described in this paper will be excluded from the meta-analysis. The resulting meta-analysis will be reported according to the PRISMA (Preferred Reporting Items for Systematic Reviews and Meta-Analyses).

### Ethical Considerations

Each member study will have obtained informed consent from participants and had their study approved by institutional ethics review boards. Full details for ethics for each study can be found in previous studies [44-65] and a summary is provided below. This study is registered in the Open Science Framework (osf.io/zbg6x).

## Results

As of August 2024, 18 longitudinal studies have agreed to participate in the GLAD project (Table 3). These studies have sample sizes ranging from 639 participants to 171,000 participants (Table 4).

The GLAD project comprises 3 essential phases, which will be repeated for other lifestyle exposures of interest: (1) conducting a systematic search to identify relevant studies and recruit study leads to contribute data, (2) generating estimates of study-level associations of dietary risk factors with CMDs using harmonized data analysis protocols developed by the GLAD Taskforce and approved by the GBD, and (3) pooling results from each study to provide the GBD with robust estimates to calculate the diet-CMD risk-outcome pairs in the first instance.

**Figure 3:**
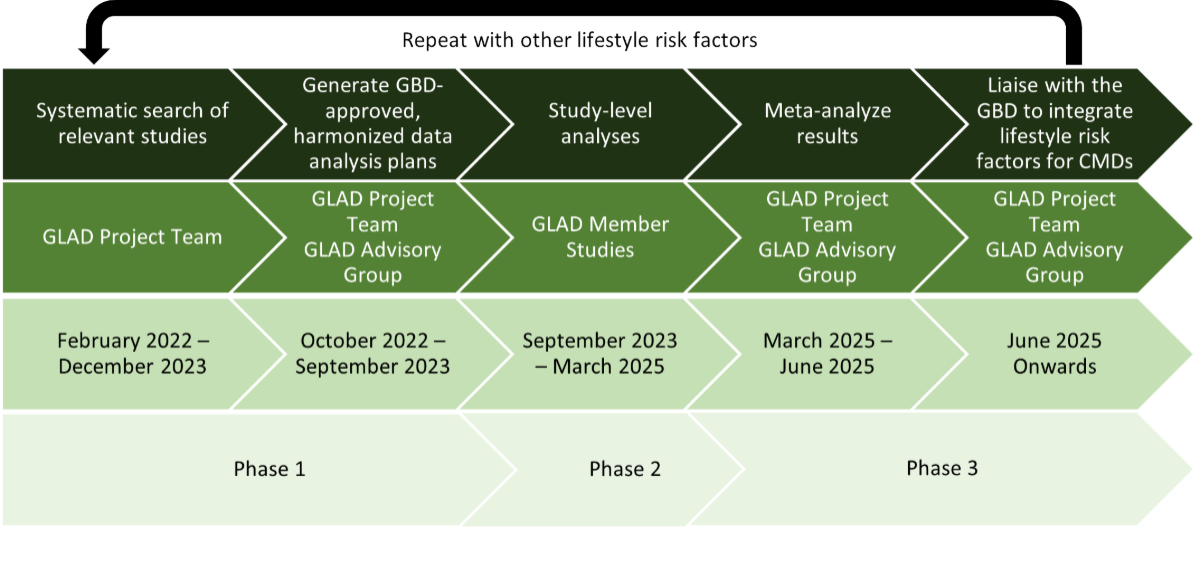
The tasks, responsible parties, phases, and expected timeline of the GLAD project. CMD: common mental disorder; GBD: Global Burden of Diseases, Injuries, and Risk Factors Study; GLAD: Global burden of disease Lifestyle And mental Disorders.

As of August 2024, phase 1 of the GLAD project has been completed, and phase 2 is due for completion in early 2025, at which time phase 3 of the GLAD project is due to begin (Figure 3).

Phase 1 includes searching for and recruiting studies with suitable data, harmonizing data definitions by ensuring member studies use the exposure definitions listed in Table 1 and the outcome definitions listed in Table 2, and harmonizing data analyses by ensuring member studies follow the statistical analysis plan presented in this paper.

**Table 3:** Details of the ethics approval for currently listed member studies^a^.

Study name	Ethics review board
The African-PREDICT^b^ study [44]	North West Department of Health and Health Research Ethics Committee of the North-West University.
Child to Adult Transition Study [45]	Royal Children’s Hospital Human Research Ethics Committee.
Dunedin Study [46]	Health and Disability Ethics Committee, Ministry of Health.
Environmental Risk (E-Risk) Longitudinal Twin Study [47,48]	Joint South London and Maudsley and the Institute of Psychiatry Research Ethics Committee.
Fragility in the Elderly Lombardy Study [49]	Ethics committee of the University of Pavia.
Geelong Osteoporosis Study [50]	Barwon Health Human Research Ethics Committee.
Health4Life [51,52]	University of Sydney, NSW^c^ Department of Education, University of Queensland, Curtin University, and relevant Catholic school ethics committees.
Healthy Life in an Urban Setting [53]	Ethical Review Board of the Academic Medical Center Amsterdam.
Longitudinal Aging Study Amsterdam [54]	Medical ethics committee of the VU University Medical Center.
Lothian Birth Cohort 1936 [55,56]	Multi-Centre Research Ethics Committee for Scotland and Lothian Research Ethics Committee.
Melbourne Collaborative Cohort Study [57]	Cancer Council Victoria's Human Research Ethics Committee.
Moli-sani Study [58]	Ethical committee of the Catholic University in Rome.
Netherlands Study of Depression and Anxiety [59]	Medical Ethical Committee of the Vrije Universiteit (VU) Medical Centre and medical ethical committees of the participating universities.
Northern Ireland Cohort of Longitudinal Ageing [60]	School of Medicine, Dentistry and Biomedical Sciences of Queen’s University Belfast.
NutriNet Brasil [61]	Ethics committee of the School of Public Health from São Paulo University
NutriNet-Santé [62]	Ethics committee of the French National Institute for Health and Medical Research and by the National Commission on Informatics and Liberty.
Piccolipiù/Piccoli+/Piccolipiù in Forma [63]	Ethics committees of the Local Health Unit Roma E (management center), of the Istituto Superiore di Sanità (National Institute of Public Health) and of each local center.
REgistre GIroní del COR [64,65]	Institut Municipal d'Assistencia Sanitaria Ethics Committee

^a^Additional studies may be participating in or providing data for the GLAD project and not be listed here.

^b^African-PREDICT: African Prospective study on the Early Detection and Identification of Cardiovascular disease and Hypertension.

^c^NSW: New South Wales.

**Table 4:** Overview of the studies participating in the GLAD^a^ project.

Study name	Study location	Maximum possible sample size^b^, n	Age range
The African-PREDICT^c^ Study [44]	South Africa	1202	20-30 years
Child to Adult Transition Study [45]	Australia	1239	Older than 8 years
Dunedin Study [46]	New Zealand	1037	0-45 years
Environmental Risk (E-Risk) Longitudinal Twin Study [47,48]	United Kingdom	2232	0-18 years
Fragility in the Elderly Lombardy Study [49]	Italy	639	Older than 65 years
Geelong Osteoporosis Study [50]	Australia	1518	Older than 30 years
Health4Life [51,52]	Australia	6639	11-13 years; 14-17 years
Healthy Life in an Urban Setting [53]	The Netherlands	24,789	18-70
Longitudinal Aging Study Amsterdam [54]	The Netherlands	3805	55-85 years
Lothian Birth Cohort 1936 [55,56]	United Kingdom	1091	Older than 60 years
Melbourne Collaborative Cohort Study [57]	Australia	41,500	Older than 40 years
Moli-sani Study [58]	Italy	24,325	Older than 35 years
Netherlands Study of Depression and Anxiety [59]	The Netherlands	3348	18-65 years
Northern Ireland Cohort of Longitudinal Ageing [60]	United Kingdom	8500	Older than 50 years
NutriNet Brasil [61]	Brazil	109,245	Older than 18 years
NutriNet-Santé [62]	France	171,000	Older than 18 years
Piccolipiù/Piccoli+/Piccolipiù in Forma [63]	Italy	3328	0-4 years
REgistre GIroní del COR [64,65]	Spain	11,158	Older than 26 years

^a^GLAD: Global burden of disease Lifestyle And mental Disorders.

^b^Maximum possible sample size refers to the largest reported sample size from study publications, study websites study protocol, or profile papers. The actual sample size to be included in the GLAD project may vary.

^c^African-PREDICT: African Prospective study on the Early Detection and Identification of Cardiovascular disease and Hypertension.

## Discussion

### Anticipated Impact

The GLAD project is a global collaboration designed to estimate the risk of CMDs attributable to lifestyle risk factors, with this protocol focusing on dietary risk factors. The anticipated results will provide the GBD with the required threshold of evidence for lifestyle risks to be causally related to CMDs, thereby enabling lifestyle-CMDs risk-outcome pairs to be integrated into the GBD framework. Specifically, the GLAD project will demonstrate that GBD-defined lifestyle risks (eg, diets low in fruit) will be associated with increased risk of CMDs. The GBD study is the largest and most comprehensive global epidemiological health study, providing metrics for 369 diseases and injuries and 87 risk factors [19,66]. These health metrics, including estimates such as the burden of disease and disability-adjusted life years, are publicly available via the GBD digital platform [13] and provide critical information for clinicians, researchers, and policy makers worldwide. While lifestyle risk factors, including dietary intake defined as per GBD-specific definitions, have been considered in the context of chronic physical conditions in the GBD study, they have not yet been linked with mental health outcomes. As the first global collaborative study to link these lifestyle risks with CMDs, using the definitions and methods concordant with the GBD, the results of the GLAD project can be used to link these lifestyle factors as risks for CMDs in the GBD. This effort will allow us to quantify the potential reduction or elimination of the CMD burden by targeting these risk factors at regional and global levels, and to ensure these data are available freely online via the GBD VizHub Data visualization tool.

Risk factors for CMDs in the GBD are currently limited, and so, the inclusion of these additional lifestyle risk factors will represent a significant expansion in the scope of the GBD, which will have global policy implications. Our work builds upon the previous efforts of studies that have successfully integrated novel risk factors for CMDs in the GBD. For example, bullying victimization has been successfully incorporated into the GBD as a level 3 risk and is now routinely integrated into GBD papers [67,68]. By emulating the successful integration of these other risk factors into the GBD, our comprehensive, collaborative approach to harmonized statistical analyses across multiple, internationally renowned epidemiological cohorts has the potential to establish a basis for evidence-backed preventive strategies focused on lifestyle risk factors to reduce the burden of CMDs. Furthermore, by operationalizing the definitions to be in line with the GBD, which are used to inform global policy guidelines, and by capturing data from different regions, sexes, and ages, these results have the potential to bring benefits to individuals and communities on a global scale.

There is substantial observational [69-71], intervention [72-76], and meta-analytical [5,77-79] evidence linking these lifestyle factors to CMDs globally, which suggests that lifestyle factors are potentially suitable (and importantly, modifiable) preventive strategies. This has been recognized by the Royal Australian and New Zealand College of Psychiatrists, with lifestyle treatments integrated into the recent clinical practice guidelines for mood disorders, including depressive disorders [9]. A 2020 meta-review identified that targeting lifestyle variables like physical activity and smoking can be used effectively in the prevention and treatment of CMDs [5]. However, there was less evidence for the role of targeting diet in preventing CMDs, particularly as defined by international policy guidelines or as per the GBD framework. In order to incorporate additional lifestyle factors into guidelines globally, we need comprehensive epidemiological evidence, with a particular focus on dietary risks. The GLAD project will address previous methodological limitations through global collaborations and following a rigorous methodological framework informed by the GBD.

Although mechanistic evidence is currently limited, the causal role of lifestyle risks for CMDs has been supported by shared biological mechanisms between lifestyle factors and CMDs. These pathways include inflammation, oxidative stress, epigenetics, hypothalamic-pituitary-adrenal axis regulation, and the gut microbiome [80]. Inflammation has been linked to CMDs previously, and physical activity, diet, sleep, and smoking have all been shown to impact inflammation [5]. As such, improving dietary intake, increasing physical activity, improving sleep, and smoking cessation may improve mental health via anti-inflammatory properties. Another mechanistic pathway shared by CMDs and many lifestyle risk factors (including diet, physical activity, sleep, and smoking) is the microbiome [80,81]. Many of the dietary risks listed in the GBD, including fruit, vegetable, whole grain, and fiber intake, have pre- and probiotic potential which has been shown to beneficially influence the gut microbiome, and other GBD-defined dietary risks, such as processed meats and sugar-sweetened beverages (which are markers for a high-fat diet), Western-style diet (have been shown to increase anxiety and depression via gut microbiome alterations) [80]. Despite the evidence linking lifestyle risk factors with CMDs, including studies identifying potential mechanisms of action, studies assessing the relationship between lifestyle risks as defined by the GBD with CMD outcomes are lacking. Further, to integrate lifestyle risks for CMDs in the GBD, evidence needs to be generated globally, and not just from individual studies. As a global collaborative project, the results from the GLAD project will therefore provide the necessary evidence to incorporate lifestyle as a risk factor for CMDs in the GBD, which will enable policy makers around the world to make policy decisions regarding the potential public health benefit of population-level lifestyle improvements to mental health.

### Strengths and Limitations

Currently, the GBD is unable to include lifestyle exposures such as dietary intake as a risk for CMDs due to the following: difficulties determining directionality and causality, inconsistency in dietary variables and methods, small sample sizes, and lack of global representation. A strength of the GLAD project is that it will address all these methodological considerations. We will (1) prioritize prospective studies using incident cases (ie, excluding those with baseline CMDs) to obtain risks of CMDs attributable to dietary intake, and not the other way around (to determine directionality or temporality), (2) include all study designs (including from experimental or randomized controlled trials to strengthen causal conclusions), (3) conduct harmonized data analyses approved by the GBD (to improve consistency between studies and specificity of the diet-CMD relationship), and (4) pool data from multiple studies from around the world (to address sample size and global representation).

Despite the clear strengths of the GLAD project, there are some limitations. First, the GBD study is an ongoing and dynamic study, and definitions and methods are constantly evolving. Every effort will be made to ensure the taskforce are following GBD-approved methods, however, some changes from the GBD may be unavoidable. Second, since the GBD does not currently include ultraprocessed food as a dietary risk factor, the definitions and methods used by the task force may not reflect those used in future iterations of the GBD. Finally, although food intakes provide a general overview of diet quality, people do not eat food items in isolation. The current methods used by the GBD do not currently consider dietary patterns. This may limit interpretations to specific food items and not to an overall dietary pattern.

### Conclusion

This multicountry, 5-year project has the potential to highlight the role of modifiable lifestyle risk factors in the prevalence and incidence of CMDs. Given the global burden of depression and anxiety, new approaches and targets for their prevention and management are of unprecedented importance.

## Appendix

Multimedia Appendix 1 Commonly used tools and scales for assessing common mental disorders, and their cut-offs for indicative diagnosis.

## Abbreviations

CMD: common mental disorder
GBD: Global Burden of Diseases, Injuries, and Risk Factors Study
GLAD: Global burden of disease Lifestyle And mental Disorders
PRISMA: Preferred Reporting Items for Systematic Reviews and Meta-Analyses

## Data Availability

The GLAD project is a multicountry collaborative effort for which individual collaborators apply a harmonized protocol developed by the task force to their own data. Data are not pooled in a shared repository or location, nor publicly available. The data custodians of each dataset may or may not make their data available upon request.
