# Supplementary material for: Estimating the Burden of Common Mental Disorders Attributable to Lifestyle Factors: Protocol for the Global Burden of Disease Lifestyle and Mental Disorder (GLAD) Project
Source: JMIR Res Protoc. 2025 Mar 14;14:e65576. doi: 10.2196/65576 (PMC11953606; doi:10.2196/65576)
Supplement: Multimedia Appendix 1 [file resprot_v14i1e65576_app1.docx]

**Table S1:** Commonly used tools and scales for assessing common mental disorders, and their cut-offs for indicative diagnosis.

| **Tool** | **Cut-Off/Details** |
| --- | --- |
| Anxiety and Related Disorders Interview Schedule (ADIS) [1] | Structed diagnostic interview |
| Beck Anxiety Inventory (BAI) [2] | ≥16 [3] |
| Beck Depression Inventory – Second Edition (BDI-II) [4] | ≥16 [5] |
| Center for Epidemiologic Studies Depression Scale (CES-D) [6] | ≥16 [6] |
| Child Behavior Checklist (CBCL) [7] | T-score ≥70 [8] |
| Clinical Interview Schedule – revised (CIS-R) [9] | Structured interview |
| Depression Anxiety Stress Scales (DASS) [10] | Anxiety subscale ≥15  Depression subscale ≥21 [11] |
| Depression Anxiety Stress Scales Short Form Version (DASS-21) [10] | Anxiety subscale ≥5  Depression subscale ≥12 [12] |
| Depression Anxiety Stress Scale – 10 (DASS-10) [13] | >7 [13] |
| Diagnostic Interview for Anxiety, Mood, Obsessive-Compulsive and Related Neuropsychiatric Disorders [14] | Semi-structed diagnostic interview |
| Distress Questionnaire 5 (DQ5) [15] | ≥11 (most disorders)  ≥14 (clinical case finding) [15] |
| General Health Questionnaire (GHQ) [16] | GHQ-60: ≥12  GHQ-30: ≥5  GHQ-28: ≥9 (anxiety subscale)  GHQ-28: ≥3 (depression subscale)  GHQ-12: ≥2 [17] |
| Generalized Anxiety Disorder (GAD) [18] | GAD-7: ≥10 [18]  GAD-2: ≥3 [19] |
| Geriatric Depression Scale (GDS) [20] | ≥11 [20] |
| Hamilton Anxiety Rating Scale (HAM-A or HARS) [21] | Interview-based; ≥13 [22] |
| Hamilton Rating Scale for Depression or the Hamilton Depression Rating Scale (HAM-D, HRSD or HDRS) [23] | Interview-based; ≥20 [23] |
| Hospital Anxiety and Depression Scale (HADS) [24] | ≥8 for each sub-scale [24] |
| Kessler Psychological Distress Scale (K10) [25] | ≥20 [26,27] |
| Major Depression Inventory (MDI) [28] | ≥21 [28,28] |
| Malaise Inventory [29] | ≥5 on the 15-item scale  ≥3 on the 9-item scale [29] |
| MINI International Neuropsychiatric Interview (MINI) [30] | Structed diagnostic interview |
| Montgomery-Asberg Depression Rating Scale (MADRS) [31] | Interview-based; ≥18 [32] |
| Mood and Feelings Questionnaire (MFQ) [33] | ≥28 [34] |
| Patient Health Questionnaire Depression Scale (PHQ) [35–37] | PHQ-9 ≥10 [35] PHQ-8 ≥10 [36] PHQ-2 ≥3 [37] |
| Quick Inventory of Depression Symptomatology – Clinician rating (QIDS-C) and Patient Rating (QIDS-P) [38] | Interview-based; ≥13 [39] |
| Short Mood and Feelings Questionnaire (SMFQ) [40] | ≥12 [41] |
| Strengths and Difficulties Questionnaire (SDQ) [42] | Emotion sub-scale: ≥5 for anxiety and depression Hyperactivity sub-scale: ≥10 for anxiety and depression [43] |
| Structured Clinical Interview for DSM-5 (SCID-5) [44] | Structured diagnostic interview |
| State Trait Anxiety Inventory-State (STAI-S) and State Trait Anxiety Inventory-Trait (STAI-T) [45] | STAI-S ≥39 STAI-T ≥52 [46] |
| World Health Organization World Mental Health Composite International Diagnostic Interview (CIDI) [47] | Structured diagnostic interview |
| 5-item World Health Organization Well-Being Index (WHO-5) [48] | <13 [48] |

This is not an exhaustive list of available tools to assess depression or anxiety. Additional tools and scales may be used within the GLAD Taskforce provided a validated cut-off score can be used.

**References**

1. Brown TA, Barlow DH. Anxiety and Related Disorders Interview Schedule for DSM-5 (ADIS-5) - Adult Version: Client Interview Schedule 5-Copy Set. 1st ed. Oxford, England: Oxford University Press; 2014.
2. Beck AT, Epstein N, Brown G, et al. An inventory for measuring clinical anxiety: psychometric properties. J Consult Clin Psychol. 1988;56(6):893-897.
3. Bardhoshi G, Duncan K, Erford BT. Psychometric meta-analysis of the English version of the Beck Anxiety Inventory. J Couns Dev. 2016;94(3):356-373.
4. Beck AT, Steer RA, Brown G. Beck Depression Inventory–II [published online 1996]. APA PsycTests. (doi: 10.1037/t00742-000).
5. Sprinkle SD, Lurie D, Insko SL, et al. Criterion validity, severity cut scores, and test-retest reliability of the Beck Depression Inventory-II in a university counseling center sample. J Couns Psychol. 2002;49:381-385.
6. Radloff LS. The CES-D Scale: A self-report depression scale for research in the general population. Appl Psychol Meas. 1977;1:385-401.
7. Achenbach TM, Ruffle TM. The Child Behavior Checklist and related forms for assessing behavioral/emotional problems and competencies. Pediatr Rev. 2000;21(8):265-271.
8. Petty CR, Rosenbaum JF, Hirshfeld-Becker DR, et al. The child behavior checklist broad-band scales predict subsequent psychopathology: A 5-year follow-up. J Anxiety Disord. 2008;22(3):532-539.
9. Lewis G, Pelosi AJ, Araya R, et al. Measuring psychiatric disorder in the community: A standardized assessment for use by lay interviewers. Psychol Med. 1992;22:465-486.
10. Lovibond SH, Lovibond PF. Manual for the Depression Anxiety Stress Scales. 2nd ed. Sydney, Australia: Psychology Foundation; 1995.
11. Beaufort IN, De Weert-Van Oene GH, Buwalda VAJ, et al. The Depression, Anxiety and Stress Scale (DASS-21) as a screener for depression in substance use disorder inpatients: A pilot study. Eur Addict Res. 2017;23(5):260-268.
12. Nieuwenhuijsen K, de Boer AGEM, Verbeek JHAM, et al. The Depression Anxiety Stress Scales (DASS): detecting anxiety disorder and depression in employees absent from work because of mental health problems. Occup Environ Med. 2003;60 Suppl 1(Suppl 1):i77-82.
13. Kim Halford W, Frost ADJ. Depression Anxiety Stress Scale-10: A brief measure for routine psychotherapy outcome and progress assessment. Behav Change. 2021;38(4):221-234.
14. Tolin DF, Gilliam C, Wootton BM, et al. Psychometric properties of a structured diagnostic interview for DSM-5 anxiety, mood, and obsessive-compulsive and related disorders. Assessment. 2018;25(1):3-13.
15. Batterham PJ, Sunderland M, Carragher N, et al. The Distress Questionnaire-5: Population screener for psychological distress was more accurate than the K6/K10. J Clin Epidemiol. 2016;71:35-42
16. Goldberg D. The Detection of Psychiatric Illness by Questionnaire. Oxford, United Kingdom: Oxford University Press; 1972.
17. Van Hemert AM, Den Heijer M, Vorstenbosch M, et al. Detecting psychiatric disorders in medical practice using the General Health Questionnaire. Why do cut-off scores vary? Psychol Med. 1995;25(1):165-170.
18. Spitzer RL, Kroenke K, Williams JBW, et al. A brief measure for assessing generalized anxiety disorder: The GAD-7. Arch of Intern Med. 2006;166(10):1092-1097.
19. Kroenke K, Spitzer RL, Williams JBW, et al. Anxiety disorders in primary care: Prevalence, impairment, comorbidity, and detection. Ann Intern Med. 2007;146(5):317-325.
20. Yesavage JA, Brink TL, Rose TL, et al. Development and validation of a geriatric depression screening scale: A preliminary report. J Psych Res. 1982;17(1):37-49.
21. Hamilton M. The assessment of anxiety states by rating. Br J Med Psychol. 1959;32(1):50-55.
22. Leentjens AFG, Dujardin K, Marsh L, et al. Anxiety rating scales in Parkinson’s disease: A validation study of the Hamilton anxiety rating scale, the Beck anxiety inventory, and the hospital anxiety and depression scale. Mov Disord. 2011;26(3):407-415.
23. Hamilton M. A rating scale for depression. J Neurol Neurosurg Psychiatry. 1960;23(1):56.
24. Zigmond AS, Snaith RP. The Hospital Anxiety and Depression Scale. Acta Psychiatr Scand. 1983;67(6):361-370.
25. Kessler RC, Andrews G, Colpe LI, et al. Short screening scales to monitor population prevalences and trends in non-specific psychological distress. Psychol Med. 2002;32(6):959-976.
26. Andrews G, Slade T. Interpreting scores on the Kessler Psychological Distress Scale (K10). Aust N Z J Public Health. 2001;25(6):494-497.
27. Hodge A, Almeida OP, English DR, et al. Patterns of dietary intake and psychological distress in older Australians: Benefits not just from a Mediterranean diet. Int Psychogeriatr. 2013;25(3):456-466.
28. Bech P, Jensen DV, Martiny K, et al. The internal and external validity of the Major Depression Inventory in measuring severity of depressive states. Psychol Med. 2003;33(2):351-356.
29. Rodgers B, Pickles A, Power C, et al. Validity of the Malaise Inventory in general population samples. Soc Psychiatry Psychiatr Epidemiol. 1999;34(6):333-341.
30. Sheehan DV, Lecrubier Y, Sheehan KH, et al. The Mini-International Neuropsychiatric Interview (M.I.N.I.): The development and validation of a structured diagnostic psychiatric interview for DSM-IV and ICD-10. J Clin Psychiatry. 1998;59 Suppl 20:22-33;quiz 34-57.
31. Montgomery SA, Åsberg M. A new depression scale designed to be sensitive to change. Br J Psychiatry. 1979;134(4):382-389.
32. Williams JBW, Kobak KA. Development and reliability of a structured interview guide for the Montgomery-Åsberg Depression Rating Scale (SIGMA). Br J Psychiatry. 2008;192(1):52-58.
33. Costello EJ, Angold A. Scales to assess child and adolescent depression: checklists, screens, and nets. J Am Acad Child Adolesc Psychiatry. 1988;27(6):726-737.
34. Thabrew H, Stasiak K, Bavin LM, et al. Validation of the Mood and Feelings Questionnaire (MFQ) and Short Mood and Feelings Questionnaire (SMFQ) in New Zealand help-seeking adolescents. Int J Methods Psychiatr Res. 2018;27(3):e1610.
35. Kroenke K, Spitzer RL, Williams JB. The PHQ-9: validity of a brief depression severity measure. J Gen Intern Med. 2001;16(9):606-613.
36. Wu Y, Levis B, Riehm KE, et al. Equivalency of the diagnostic accuracy of the PHQ-8 and PHQ-9: A systematic review and individual participant data meta-analysis. Psychol Med. 2020;50(8):1368-1380.
37. Kroenke K, Spitzer RL, Williams JBW. The Patient Health Questionnaire-2: Validity of a two-item depression screener. Med Care. 2003;41(11):1284-1292.
38. Rush AJ, Trivedi MH, Ibrahim HM, et al. The 16-Item quick inventory of depressive symptomatology (QIDS), clinician rating (QIDS-C), and self-report (QIDS-SR): A psychometric evaluation in patients with chronic major depression. Biol Psychiatry. 2003;54(5):573-583.
39. Lamoureux BE, Linardatos E, Fresco DM, et al. Using the QIDS-SR16 to Identify Major Depressive Disorder in Primary Care Medical Patients. Behav Ther. 2010;41(3):423-431.
40. Angold A, Costello EJ, Messer SC, et al. Development of a short questionnaire for use in epidemiological studies of depression in children and adolescents. Int J of Methods Psychiatr Res. 1995;5:237-249.
41. Eyre O, Bevan Jones R, Agha SS, et al. Validation of the short Mood and Feelings Questionnaire in young adulthood. J Affect Disord. 2021;294:883-888.
42. Goodman R. The Strengths and Difficulties Questionnaire: A research note. J Child Psychol Psychiatry. 1997;38(5):581-586.
43. Bryant A, Guy J, Holmes J. The Strengths and Difficulties Questionnaire predicts concurrent mental health difficulties in a transdiagnostic sample of struggling learners. Front Psychol. 2020;11:587821.
44. First MB, Williams JBW, Karg RS, et al. User’s Guide for the SCID-5-CV Structured Clinical Interview for DSM-5® Disorders: Clinical Version. Washington, DC: American Psychiatric Publishing; 2016.
45. Spielberger CD, Gorssuch RL, Lushene PR, et al. Manual for the State-Trait Anxiety Inventory. Washington, DC: Consulting Psychologists Press; 1983.
46. Dennis RE, Boddington SJA, Funnell NJ. Self-report measures of anxiety: Are they suitable for older adults? Aging Ment Health. 2007;11(6):668-677.
47. Kessler RC, Üstün TB. The World Mental Health (WMH) Survey Initiative version of the World Health Organization (WHO) Composite International Diagnostic Interview (CIDI). Int J Methods Psychiatr Res. 2004;13(2):93-121.
48. World Health Organization. Wellbeing Measures in Primary Health Care/the DepCare Project: Report on a WHO Meeting. Stockholm, Sweden: World Health Organization; 1998 (https://apps.who.int/iris/handle/10665/349766).
